# Application of Efficient Data Cleaning Using Text Clustering for Semistructured Medical Reports to Large-Scale Stool Examination Reports: Methodology Study

**DOI:** 10.2196/10013

**Published:** 2019-01-08

**Authors:** Hyunki Woo, Kyunga Kim, KyeongMin Cha, Jin-Young Lee, Hansong Mun, Soo Jin Cho, Ji In Chung, Jeung Hui Pyo, Kun-Chul Lee, Mira Kang

**Affiliations:** 1 Department of Digital Health Samsung Advanced Institute for Health Sciences & Technology Sungkyunkwan University Seoul Republic of Korea; 2 Statistics and Data Center Research Institute for Future Medicine Samsung Medical Center Seoul Republic of Korea; 3 Center for Health Promotion Samsung Medical Center Sungkyunkwan University School of Medicine Seoul Republic of Korea; 4 Jason TG Seoul Republic of Korea

**Keywords:** data cleaning, text clustering, key collision, nearest neighbor methods, OpenRefine

## Abstract

**Background:**

Since medical research based on big data has become more common, the community’s interest and effort to analyze a large amount of semistructured or unstructured text data, such as examination reports, have rapidly increased. However, these large-scale text data are often not readily applicable to analysis owing to typographical errors, inconsistencies, or data entry problems. Therefore, an efficient data cleaning process is required to ensure the veracity of such data.

**Objective:**

In this paper, we proposed an efficient data cleaning process for large-scale medical text data, which employs text clustering methods and value-converting technique, and evaluated its performance with medical examination text data.

**Methods:**

The proposed data cleaning process consists of text clustering and value-merging. In the text clustering step, we suggested the use of key collision and nearest neighbor methods in a complementary manner. Words (called values) in the same cluster would be expected as a correct value and its wrong representations. In the value-converting step, wrong values for each identified cluster would be converted into their correct value. We applied these data cleaning process to 574,266 stool examination reports produced for parasite analysis at Samsung Medical Center from 1995 to 2015. The performance of the proposed process was examined and compared with data cleaning processes based on a single clustering method. We used OpenRefine 2.7, an open source application that provides various text clustering methods and an efficient user interface for value-converting with common-value suggestion.

**Results:**

A total of 1,167,104 words in stool examination reports were surveyed. In the data cleaning process, we discovered 30 correct words and 45 patterns of typographical errors and duplicates. We observed high correction rates for words with typographical errors (98.61%) and typographical error patterns (97.78%). The resulting data accuracy was nearly 100% based on the number of total words.

**Conclusions:**

Our data cleaning process based on the combinatorial use of key collision and nearest neighbor methods provides an efficient cleaning of large-scale text data and hence improves data accuracy.

## Introduction

In all of the industries, including the medical field, complex and diverse (structured, semistructured, unstructured) data have been growing dramatically for decades [1-3]. Although most health data have been digitalized, it is still not easy to handle medical records such as examination reports or physician’s notes because they are historically based on paper records and generated data mainly in semistructured or unstructured forms. In addition, they may contain a variety of nonidentical duplicates, typographical errors, inconsistencies, and data entry problems [4-7].

High performance analysis requires clean and high-quality data to yield reliable results [8-11]. Therefore, efficient data cleaning takes precedence to improve the quality of data and obtain accurate analysis results [12]. However, researchers are commonly faced with many obstacles in transforming the data into a clean and high-quality dataset owing to diverse patterns of typographical errors and duplicates.

For text analysis of semistructured or unstructured data, we can use a paid program such as SAS Content Categorization (SAS Institute Inc) or IBM Watson Content Analytics (IBM) [13,14]. However, these programs are very expensive and are not readily available to individual researchers because they are mainly sold to companies or research groups. Also, these programs require extensive practice and experience.

Data cleaning using Excel’s “remove duplicates” function has been done before, but it is mostly unpractical to clean the data using Excel tools. Some of the nonidentical duplicates still remain because they are not recognized as duplicates when special characters or punctuations appear [5,6,15,16]. Duplicate detection tools such as the Febrl system, TAILOR, and BigMatch were also used in cleaning data. However, Febrl has usability limitations such as slowness, unclear error messages, and complicated installations [17-20]. The listed programs are rather complex to the average users who do not have experience with programming and language functions.

Many researchers who interpret and clean the local datasets are domain experts and are not familiar with the programming language [21]. Thus, researchers need user friendly cleaning tools. OpenRefine can identify all types of strings and remove duplicates without the difficulties of programming and is a free, open source tool. OpenRefine contains the following 2 clustering methods: key collision methods and nearest neighbor methods. We proposed a data cleaning process using both text clustering methods in OpenRefine to improve accuracy of semistructured data.

## Methods

**Figure 1:**
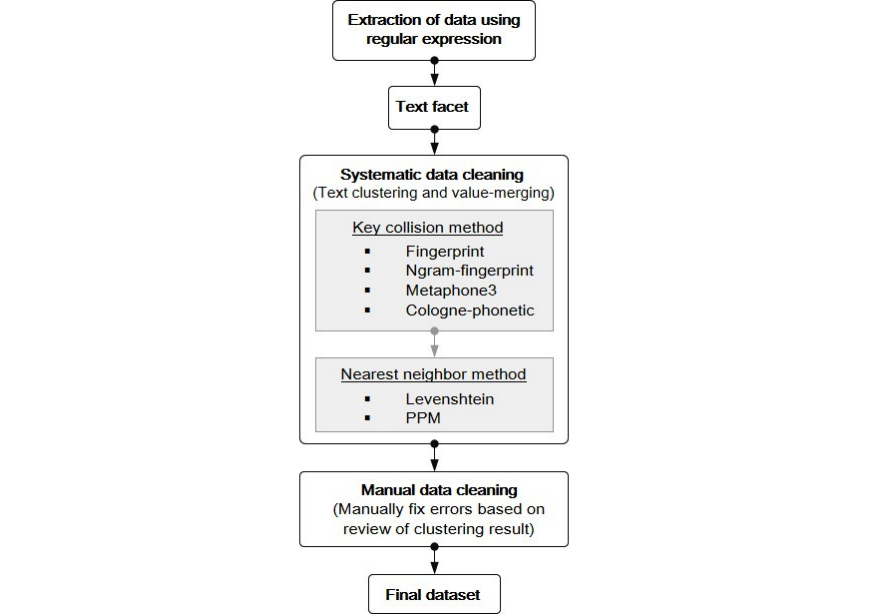
Flow chart of our data cleaning process. PPM: prediction by partial matching.

We performed data cleaning of 574,266 stool examination reports conducted at Samsung Medical Center from 1995 to 2015. Data for this study were extracted from DARWIN-C, the clinical data warehouse of Samsung Medical Center. According to the data cleaning process proposed in Figure 1, we conducted data cleaning by clustering and merging parasite names and investigated its performance.

As described in Figure 1, the proposed data cleaning process consists of the following 4 steps: preprocess, text facet, systematic cleaning, and manual cleaning. In the preprocess, only names related to parasites (ie, helminth or protozoa) in raw text data were extracted using the regular expression functions of STATA MP 14.2 version [22]. The extracted words were then uploaded on OpenRefine 2.7. In the text facet step, the number of occurrences was browsed for each word.

The systematic cleaning step consists of text clustering and value-merging. Two clustering methods (ie, key collision and nearest neighbor) are used in a complementary manner to identify word clusters, each of which is expected to contain a correct word and its wrong representations with diverse forms of typographical errors (called “wrong values”). Key collision methods work by creating an alternate representation of a key that contains only the most significant or meaningful parts of a string and by clustering different strings together based on the same key. Because key collision methods are fast and simple in a variety of contexts, they have been often used for text clustering. We sequentially used 4 key collision methods including fingerprint, N-gram fingerprint, Metaphone3, and Cologne phonetic in OpenRefine. Nearest neighbor methods (also known as kNN) are widely used for clustering as well. These methods are slower but more accurate because they calculate the distance between each value. We sequentially used two nearest neighbor methods, the Levenshtein distance method and Prediction by Partial Matching method in OpenRefine. We combined both methods to enhance the accuracy [23].

**Figure 2:**
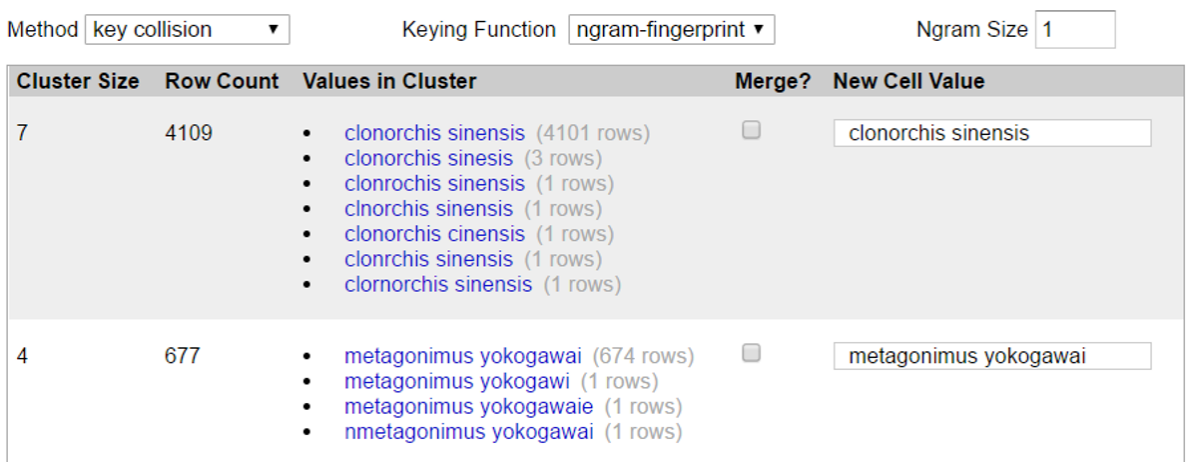
Representative screenshot of OpenRefine interface used for value-merging task.

For each identified cluster, the wrong values are converted to their correct word by value-merging. Because OpenRefine provides a convenient user interface that lists the correct word and its wrong values in each cluster in descending order of occurrence frequency, researchers can easily recognize the correct word and conduct the value-merging task. For “*Clonorchis sinensis*” in stool examination report data, a variety of wrong expressions were noticed in the same cluster, such as clonorchis sinesis, clnorchis sinensis, clonorchis cinensis, clonrchis sinensis, and clornorchis sinensis (Figure 2). By looking at the word list, we were able to efficiently choose “*Clonorchis sinensis*” as the correct word and make a quick decision to convert all the others to “*Clonorchis sinensis*” In the final step, the remaining words that did not belong to any cluster were investigated and manually cleaned when necessary.

## Results

A total of 1,167,104 words in 574,266 stool examination reports were surveyed, and words not related to the names of helminth or protozoa were excluded from the study. We discovered 30 correct words and 45 patterns of typographical errors and duplicates (Multimedia Appendix 1). The key collision methods were able to cluster the patterns of typographical errors and duplicates with the correct word except for 6 patterns. The nearest neighbor methods were able to cluster the patterns of typographical errors and duplicates with the correct word except for 2 patterns (Table 1).

**Table 1:** List of typographical errors that could not be clustered with the correct word by each method.

Correct word	Typographical error	Key collision	Nearest neighbor
Negative	Native	✗^a^	✗
	Negaitve	✓^b^	✗
Endolimax	Eolimax	✗	✓
	Endolix	✗	✓
Entamoeba	Etamoeba	✗	✓
Lamblia	Lamdlia	✗	✓
	G.lamblia	✗	✓

^a^✗: Typographical error is not clustered with correct word.

^b^✓: Typographical error is clustered with correct word.

**Table 2:** Correction rates by each method.

Method	Correction rate by the number of typographical error patterns^a^, %	Correction rate by the number of typographical error words^b^, %
Key collision	86.67	91.67
Nearest neighbor	95.56	97.22
Using both	97.78	98.61

^a^The number of corrected typographical error patterns divided by the total number of typographical error patterns multipled by 100 (%).

^b^The number of corrected typographical error words divided by the total number of typographical error words multipled by 100 (%).

The word “native” was the only pattern not clustered as “negative” out of all typographical errors by any clustering method because of the high inconsistency rate of the 2 words (2/6 characters, 33%). All typographical errors and duplicates except “native” were clustered correctly. We achieved a high correction rate of 98.61% by the number of typographical error words and 97.78% by the number of typographical error patterns when using both clustering methods (Table 2). After systematic data cleaning of 1,167,104 words, only 1 word with a typographical error remained and was revised manually. Thus, the accuracy of systematic data cleaning was nearly 100% based on the number of total words.

## Discussion

Many researchers have made great efforts to study data analytics methodology, but there have been relatively few studies on data cleaning methodology for unexpected typographical errors [24,25]. It is rare to find a report that quantitatively analyzes the performance of data cleaning methods because they are often undocumented and used in nonofficial ways [24]. In this study, we suggested an efficient way of data cleaning for large-scale medical text data and investigated its cleaning performance. Although several methods of text analysis exist, it is not easy for general researchers to use these methods. Most methods are not readily available or have limitations in usability. Therefore, there is a need for more feasible and user friendly methods for cleaning large-scale text datasets.

We employed OpenRefine for data cleaning because of the following advantages. First, individual researchers can easily access and use OpenRefine because it is a free and open source tool. Second, OpenRefine provides researchers with an easy interface to clean the data without difficulties of programming. Third, one can easily fix rare typographical errors (which are not automatically corrected) manually and have the opportunity to modify false positive clustering [6,23].

However, we still need much effort to review each clustering result and decide whether to merge, especially in cases where the number of clustering is extremely large. In addition, formal technical support for OpenRefine is not available, and it is supported by user forums or communities. Despite these limitations, OpenRefine is a useful and effective support tool for labor-intensive and time-consuming data cleaning of semistructured data.

Our data cleaning process can be applied to other types of semistructured text data because we observed that the combinatorial use of key collision and nearest neighbor methods resulted in efficient and reliable data cleaning.

## Appendix

Multimedia Appendix 1 Patterns of parasite names in stool examination reports.
