# Supplementary material for: Application of Efficient Data Cleaning Using Text Clustering for Semistructured Medical Reports to Large-Scale Stool Examination Reports: Methodology Study
Source: J Med Internet Res. 2019 Jan 8;21(1):e10013. doi: 10.2196/10013 (PMC6329435; doi:10.2196/10013)
Supplement: Multimedia Appendix 1 [file jmir_v21i1e10013_app1.pdf]

Multimedia Appendix 1. Patterns of parasite names in stool examination reports.

Helminth			Protozoa		
Patterns in raw data		Number of words	Patterns in raw data		Number of words
**Negative**[a]		567,836	**Negative**[a]		560,450
	Negaitve[b]	1		Nagative[b]	1
	Negative6[b]	1		Negativ[b]	1
	Native[b]	1	**Entamoeba**[a]		2904
**Sinensis**[a]		4225		Entameoba[b]	12
	Cinensis[b]	1		Entameba[b]	2
	Sinesis[b]	3		Entanoeba[b]	1
**Clonorchis**[a]		4223		Etamoeba[b]	1
	Clonochis[b]	2		Enatamoeba[b]	1
	Clornorchis[b]	1		Entamoba[b]	1
	Clonrochis[b]	1		Entomoeba[b]	1
	Clnorchis[b]	1	**Coli**[a]		2776
	Clonrchis[b]	1		Colie[b]	1
**Trichuris**[a]		792		Col[b]	1
	Trichurus[b]	2	**Endolimax**[a]		9664
**Trichiura**[a]		791		Endolimaxs[b]	2
	Trichura[b]	2		Edolimax[b]	1
	Trichria[b]	1		Nedolimax[b]	1
**Metagonimus**[a]		689		Nendolimax[b]	1
	Mtagonimus[b]	1		Eolimax[b]	1
	Nmetagonimus[b]	1		Endolix[b]	1
**Yokogawai**[a]		684	**Nana**[a]		9673
	Yokogawa[b]	4		Nan[b]	2
	Yokogawi[b]	1		Nanae[b]	1

	Yokogawaie[b]	1		Nanac[b]	1
	Yokogowai[b]	1	**Lamblia[a]**		870
Gymnophalloides[a]		175		Lamdlia[b]	1
Seoi[a]		175		G.lamblia[b]	1
Ascaris[a]		19	**Giardia[a]**		870
Lumbricoides[a]		19		Girdia[b]	2
Diphyllobothrium[a]		8	**Histolytica[a]**		138
Latum[a]		8		Histolytoca[b]	1
Enterobius[a]		6			
Vermicularis[a]		6			
Paragonimus[a]		4			
Westermani[a]		3			
Hookworm[a]		1			
**Hymenolepis[a]**		1			
	Hymenolepsis[b]	3			
Entamoeba[a]		1			
Nana[a]		4			
Diminuta[a]		1			
Trichostrongylus[a]		1			
Parasite[a]		9			
**Egg[a]**		6			
	Eggs[b]	3			

[a]Pattern of correct word (n=30).

[b]Typing error or duplicate (n=45).
